# Biocontrol of *Fusarium graminearum sensu stricto*, Reduction of Deoxynivalenol Accumulation and Phytohormone Induction by Two Selected Antagonists

**DOI:** 10.3390/toxins10020088

**Published:** 2018-02-20

**Authors:** Juan Palazzini, Pablo Roncallo, Renata Cantoro, María Chiotta, Nadia Yerkovich, Sofía Palacios, Viviana Echenique, Adriana Torres, María Ramirez, Petr Karlovsky, Sofía Chulze

**Affiliations:** 1 Department of Microbiology and Immunology, Faculty of Exact Sciences, National University of Río Cuarto, Route 36 Km 601, Río Cuarto, Córdoba 5800, Argentina; jpalazzini@exa.unrc.edu.ar (J.P.); rcantoro@agro.uba.ar (R.C.); mchiotta@exa.unrc.edu.ar (M.C.); nyerkovich@exa.unrc.edu.ar (N.Y.); spalacios@exa.unrc.edu.ar (S.P.); atorres@exa.unrc.edu.ar (A.T.); mramirez@exa.unrc.edu.ar (M.R.); 2 CERZOS-CONICET, Department of Agronomy, UNS–CCT CONICET Bahía Blanca, Camino de la Carrindanga Km 7, Bahía Blanca 8000, Argentina; roncallo@criba.edu.ar (P.R.); echeniq@criba.edu.ar (V.E.); 3 Molecular Phytopathology and Mycotoxin Research, Georg-August-University, Grisebachstrasse 6, 37077 Goettingen, Germany; pkarlov@gwdg.de

**Keywords:** biocontrol, durum wheat, phytohormones, wheat defense modulation

## Abstract

Fusarium head blight (FHB) is a devastating disease that causes extensive yield and quality losses to wheat and other small cereal grains worldwide. Species within the *Fusarium graminearum* complex are the main pathogens associated with the disease, *F. graminearum* *sensu stricto* being the main pathogen in Argentina. Biocontrol can be used as part of an integrated pest management strategy. Phytohormones play a key role in the plant defense system and their production can be induced by antagonistic microorganisms. The aims of this study were to evaluate the effect of the inoculation of *Bacillus velezensis* RC 218, *F. graminearum* and their co-inoculation on the production of salicylic acid (SA) and jasmonic acid (JA) in wheat spikes at different periods of time under greenhouse conditions, and to evaluate the effect of *B. velezensis* RC 218 and *Streptomyces albidoflavus* RC 87B on FHB disease incidence, severity and deoxynivalenol accumulation on *Triticum turgidum* L. var. *durum* under field conditions. Under greenhouse conditions the production of JA was induced after *F. graminearum* inoculation at 48 and 72 h, but JA levels were reduced in the co-inoculated treatments. No differences in JA or SA levels were observed between the *B. velezensis* treatment and the water control. In the spikes inoculated with *F. graminearum,* SA production was induced early (12 h), as it was shown for initial FHB basal resistance, while JA was induced at a later stage (48 h), revealing different defense strategies at different stages of infection by the hemibiotrophic pathogen *F. graminearum*. Both *B. velezensis* RC 218 and *S. albidoflavus* RC 87B effectively reduced FHB incidence (up to 30%), severity (up to 25%) and deoxynivalenol accumulation (up to 51%) on durum wheat under field conditions.

## 1. Introduction

Wheat is the cereal most consumed worldwide. Both bread wheat (*Triticum aestivum* L.) and durum wheat (*Triticum turgidum* L. var. *durum*) can be affected by *Fusarium* head blight (FHB). Within the *Fusarium graminearum* species complex, *Fusarium graminearum* sensu stricto is the main pathogen associated with FHB in Argentina. During the last 60 years, several epidemics of FHB of varying degrees of severity have occurred in Argentina. During 2012, a severe FHB outbreak occurred in the main wheat growing area, with estimated losses of up to 70% [1]. The concern of this disease is due to the reduction in grain yield and quality and the potential for contamination with mycotoxins, mainly trichothecenes such as deoxynivalenol (DON) and its acetylated derivatives 3-acetyl-deoxynivalenol (3-ADON) and 15-acetyl-deoxynivalenol (15-ADON) and deoxynivalenol 3 β–d-glucoside (DON-3-G) [2]. When humans or animals ingest food contaminated by this mycotoxin, DON can inhibit protein synthesis, cause immunomodulation, have reproductive effects and interfere with intercellular signaling [3,4].

Durum wheat, with a worldwide production of around 33 million tons, is an important small grain cereal, used for human consumption [5]. In Argentina, durum wheat is mainly used for pasta production. At the regional level, Argentina ranks second among pasta manufacturers in Latin America, behind Brazil and ahead of than Mexico. In the last few years, the national production of pasta has increased to 40% and in 2016 it reached 345,000 tons. The pasta consumption per capita was estimated as 8.95 kg year^−1^ [6]. Maximum permitted limits for deoxynivalenol in row and processed wheat have been established by the Commission Regulation and its amendments [7]. In particular, in durum wheat for human consumption the European Commission established that the maximum permitted levels of DON are 1750 µg kg^−1^ for unprocessed grains and 750 µg kg^−1^ for pasta.

Fusarium head blight is caused by infection during anthesis, which is facilitated under humid weather conditions. Different strategies are used to control the disease. The use of resistant cultivars is partially effective, but a resistant cultivar does not always show good agronomic traits. Fungicide application is another important strategy available for the management of FHB. The fungicides most widely used against FHB are triazoles such as metconazole, propiconazole, prothioconazole, and tebuconazole [8]. Although some fungicides can reduce FHB and the resulting accumulation of DON, the narrow time window for the application and the constraints of spraying technology limits the efficiency of the treatment [9,10]. Besides, the resistance of the *Fusarium* species to fungicides can reduce the effect of fungicide treatment [11]. 

Anthesis is the stage of the greatest susceptibility to FHB, as anthers are the common entry route into the plant [12]. Different biocontrol agents (BCAs) have been evaluated to reduce FHB, including bacteria of the genera *Bacillus* [13,14,15,16,17,18], *Pseudomonas* [17,19], *Streptomyces* [20] and *Lysobacter* [21]. Also, *Cryptococcus* species have shown antagonistic activity towards FHB pathogens [22,23]. Biocontrol of pathogens can be achieved through antibiosis, mycoparasitism, competition, and the induction of resistance in the host plant [24]. Recently, antagonistic activity of wheat endophytes against *F. graminearum* under in vitro assays was observed [25]. In previous studies, the biocontrol activity of *Bacillus velezensis* RC 218 and *Streptomyces albidoflavus* RC 87B under field conditions was demonstrated, but the performance of these BCA on durum wheat had not been evaluated [16,20,26].

The resistance responses of the active defense of plants against pathogens are mediated by phytohormones such as jasmonic acid (JA) and salicylic acid (SA) through a complex signaling pathways. JA-regulated mechanisms primarily trigger defense responses against necrotrophic pathogens while SA mechanisms are primarily activated by biotrophic pathogens. The mechanisms underlying resistance to necrotrophic and hemi-biotrophic pathogens are complex. The SA and JA defense pathways generally interact antagonistically in the resistance response [27]. The response of the phytohormone pathway in wheat to infection with *F. graminearum* has been evaluated [28,29] but the role of phytohormones in interactions involving pathogens, biocontrol agents and the host have been little explored [27].

The aims of this study were to evaluate the biocontrol activity of *B. velezensis* RC 218 and *S. albidoflavus* RC 87B on durum wheat to reduce FHB and the accumulation of DON and to study the roles of SA and JA in interactions between the host, antagonist (*B. velezensis* RC 218) and pathogen (*F. graminearum*).

## 2. Results

### 2.1. Jasmonic and Salicylic Acid Levels in Wheat Spikes Inoculated with Bacillus Velezensis RC 218 and Fusarium graminearum RC 276

**Figure 1:**
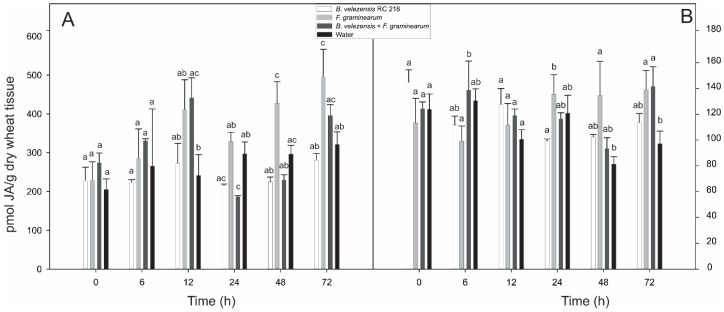
Jasmonic acid levels detected in wheat spikes after inoculation with *F. graminearum* and *B. velezensis*. (**A**) 2014/15 and (**B**) 2015/16. Different letters on each column indicate significant differences according to Tukey’s test (*p* ≤ 0.05), whiskers indicate standard deviation (SD).

**Figure 2:**
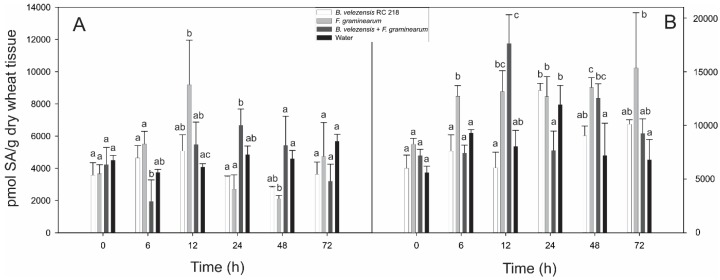
Salicylic acid levels detected in wheat spikes after inoculation with *F. graminearum* and *B. velezensis*. (**A**) 2014/15 and (**B**) 2015/16. Different letters on each column indicate significant differences according to Tukey’s test (*p* ≤ 0.05), whiskers indicate standard deviation (SD).

*Bacillus velezensis* RC 218 (Bv) and *F. graminearum* RC 276 (Fg) were inoculated individually and co-inoculated (Bv+Fg) to spikes of a susceptible wheat variety BioInta 1005 during the anthesis period and the levels of jasmonic acid (JA) and salicylic acid (SA) were evaluated at different time points after inoculation. No differences in either JA or SA levels were observed when comparing spikes treated with *B. velezensis* (Bv) and water controls in both years (*p* > 0.05) (Figure 1 and Figure 2).A significant increase of JA levels after inoculation with *F. graminearum* as compared to water controls was observed at 72 h after inoculation in the first year and at 48 h and 72 h after inoculation in the second year. Co-inoculation with Bv prevented this increase in both years (Figure 1).

Early SA production was observed at 6 and 12 h in the Fg treatment in comparison to the water control, meanwhile lower levels were detected in the Bv+Fg treatment at the same time points. The early SA production at 6 and 12 h after Fg inoculation agree with the biotrophic stage of the pathogen. At the 72 h time point, no differences in SA levels were observed in the treatments evaluated (Figure 2). No clear response between Bv+Fg and Fg treatments was observed from 12 h and later sampling time points (Figure 2).

### 2.2. Biocontrol of Fusarium graminearum by B. Velezensis RC 218 and S. Albidoflavus RC 87B on Durum Wheat under Field Trial

**Figure 3:**
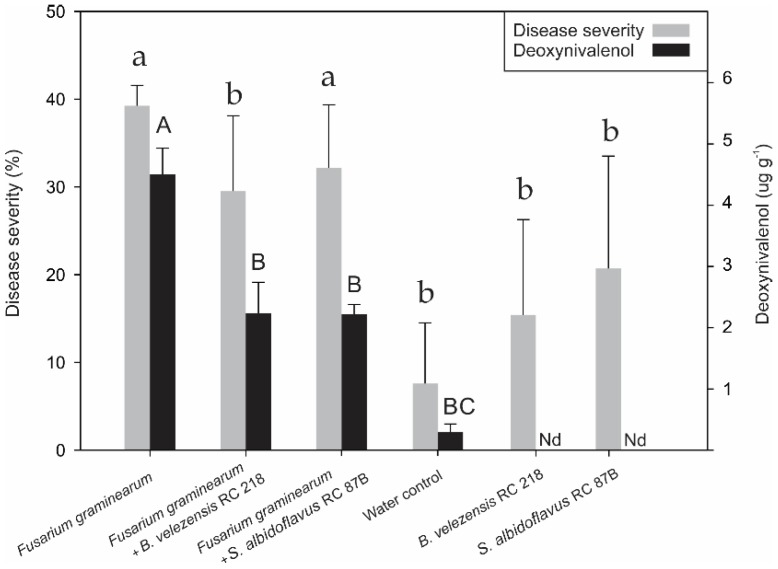
Fusarium Head Blight severity at field and deoxynivalenol accumulation on harvested grains. Disease severity means were separated using Dunn’s method (*p* ≤ 0.001); DON means were separated using Duncan’s method (*p* ≤ 0.05). Columns with different letters (lower case or capital) indicate significant differences related to disease severity or deoxynivalenol, respectively. Whiskers on the columns indicate standard deviation (SD). Nd: not detected.

The effectiveness of a formulated *B. velezensis* RC 218 product and *S. albidoflavus* RC 878B at reducing FHB incidence and severity, DON accumulation, *F. graminearum* DNA levels and *Fusarium*-damaged kernels (FDK) on harvested grains was evaluated under field trial. In the control treatment, *F. graminearum* showed 16 and 39% of FHB incidence and severity, respectively. *Bacillus velezensis* RC 218 biofungicide significantly reduced FHB incidence by 30% and FHB severity by 25% (*p* ≤ 0.05) (Figure 3). *Streptomyces albidoflavus* RC 87B reduced FHB incidence and severity at lower levels of 17 and 18%, respectively, in comparison with *B. velezensis* RC 218. About a 1.2% FHB incidence was observed in either the negative control (water inoculation) or biocontrol treatments (bacteria inoculation).

*Fusarium graminearum* DNA levels on the positive control treatment (*F. graminearum* inoculation) averaged 60.7 pg DNA mg grain^−1^. A significant reduction in DNA levels was observed when the biofungicide *B. velezensis* RC 218 was applied (60% reduction), meanwhile when *S. albidoflavus* RC 87B was applied only a 39% reduction was achieved (Table 1). In negative control plots, less than 5 pg DNA mg grain^−1^ were detected. A mean of 11% of FDK was observed in the treatment that was inoculated with *F. graminearum*. A significant reduction in the percentage of FDK was observed in the treatments where the biofungicides *B. velezensis* RC 218 and *S. albidoflavus* RC 87B where applied, with a reduction of 45 and 36%, respectively, *p* ≤ 0.05) (Table 1). 

The deoxynivalenol content in harvested grains inoculated only with *F. graminearum* (positive control) averaged 4.43 µg g^−1^. The treatments where *B. velezensis* RC 218 or *S. albidoflavus* RC 87B were applied showed a significant reduction in DON levels of 50 and 51%, respectively (Figure 3, Table 1).

## 3. Discussion

Fusarium head blight is caused by species within the *F. graminearum* complex. In Argentina, *F. graminearum sensu stricto* is the main pathogen associated with the disease [1,16]. In the pathosystem wheat–*F. graminearum*, it was demonstrated that both SA and JA signaling are associated with FHB resistance [28,29]. Bacteria and fungi can be applied to the soil or aerial parts of plants as an antagonist to improve plant growth or protect them against pathogens. These antagonist microorganisms can act through different modes of action, which can be direct, such as competition for nutrients, niche occupation, antibiosis and parasitism, or indirect by inducing resistance and plant growth promotion [24]. In a previous study, *B. velezensis* RC 218 genome mining demonstrated the presence of ten gene clusters comprising Non ribosomal peptide synthetases (NRPS) groups (Iturin A, fengycin, bacillibactin and basilysin) and polyketidesynthetase group [26]. The biocontrol activity of *B. velezensis* RC 218 could be related to the production of these secondary metabolites, which have shown antibacterial, antifungal and siderophore activities.

In the present study, we aimed to evaluate the relation between SA and JA levels in wheat spikes when *B. velezensis* RC 218 was inoculated individually and co-inoculated with the pathogen. This is the first report of the effect of a BCA that effectively reduces FHB and its interaction with *F. graminearum* and the phytohormones’ (SA and JA) signaling on wheat spikes in a susceptible wheat variety. During the first year’s experiment, *F. graminearum* applied alone induced early SA and late JA production on wheat spikes, which is in concordance with the biotrophic and necrotrophic stages of the pathogen, respectively [29]. These results agree with those observed by Ding et al. [28], who observed early (6 h) expression of genes activated by SA and late gene expression activated by JA. In our study, the co-inoculation treatment reduced SA accumulation after 6 h; meanwhile, the pathogen treatment was higher. A clear reduction in JA production was observed in the co-inoculated treatment after 24, 48 and 72 h, indicating a possible biocontrol effect on the pathogen that could reduce the plant defense response through phytohormone modulation. In contrast, Henkes et al. [30], working with the BCA *Pseudomonas fluorescens* CHA0 under in situ conditions, observed that the application of the bacteria to wheat roots induced a systemic resistance mechanism against *F. graminearum*, and concluded that the effect of the bacteria on plant defense was more important that the direct interaction of the bacteria with the pathogen. 

Durum wheat is more susceptible to FHB than bread wheat [31]. On bread wheat, we showed that *B. velezensis* RC 218 and *S. albidoflavus* RC 87B effectively reduced FHB severity and DON accumulation on bread wheat by 39–76% and 69–100%, respectively. The present study provides data on the biocontrol ability of these two biofungicides to reduce FHB parameters on a susceptible durum wheat variety. The first report of FHB biocontrol on durum wheat was done by Schisler et al. [32]. The authors observed effective antagonistic activity of bacteria and yeasts in diminishing FHB severity by up to 90%, but no effect on DON reduction was observed. Finally, they concluded that field location influenced BCA activity more than the durum wheat cultivar. *Bacillus velezensis* RC 218 and *S. albidoflavus* RC 87B showed similar biocontrol performance both in bread and durum wheat and in different locations, indicating a better adaptation of the BCAs to different environments. In the case of *B. velezensis* RC 218, this fact could be attributed to the physiological improvement (based on the accumulation of betaine) performed to the BCA prior to formulation [33]. Also, Baffoni et al. [34] evaluated the interaction of *Lactobacillus plantarum* SLG17 and *Bacillus amyloliquefaciens* FNL13 on durum wheat. Despite the authors observing low FHB severity in the different treatments, a significant FHB index reduction was observed, although they reported needing repeated applications (Zadoks growth stage from GS 50 to GS 87) to obtain biocontrol activity and the DON level after harvest was not reported.

Deoxynivalenol is the predominant trichothecene when favorable conditions led to FHB outbreaks. In Argentina, Cendoya et al. [35] reported DON occurrence in durum wheat grains during the 2011/12 wheat harvest season, with values up to 15 µg g^−1^. Additionally, in another study carried out in 2012/13 and 2013/14, Palacios et al. [2] showed a natural occurrence of DON and DON-3-Glucoside on 84 durum wheat samples, with 30% of the samples exceeding the maximum levels established by the EC [7]. From a food safety point of view, the effectiveness of the evaluated BCAs in reducing DON levels by up to 50% is very important since durum wheat is used in the human diet through pasta consumption. 

Real time PCR (qPCR) is an important tool that can be used to deduce FHB parameters that have occurred previously in the field. In our study, we found a positive correlation between qPCR and severity, incidence and DON (*p* ≤ 0.01; *R* = 0.65, 0.7 and 0.75, respectively). In addition, Horevaj et al. [36] evaluated *F. graminearum* fungal biomass by qPCR and found a positive correlation between FDK, disease severity and DON content on harvested grains and field samples. Demeke et al. [37] also found a positive correlation in quantified *F. graminearum* DNA, FDK and DON (*R*^2^ ≥ 0.83). These facts reveal the effectiveness and importance of correlating DNA levels by qPCR with FHB severity in the field and DON accumulation on harvested grains.

The wheat plant could assume that *B. velezensis* RC 218 is a beneficial microbe, without triggering any defense response, which does not happen with pathogen infection. When analyzing the bacterial-pathogen interaction, only a SA/JA modulation was observed. Further studies including gene expression analysis could clarify this situation when biocontrol, pathogen and wheat plants interact together. The biocontrol effect of *B. velezensis* RC 218 against *F. graminearum* could be mediated through antibiosis (secondary metabolite production), as it was previously evidenced [26]. Physiological improvement could be a key factor for BCA performance stability for the formulation to be used in different environments or locations. The biocontrol effectiveness of *B. velezensis* RC 218 and *S. albidoflavus* RC 87B on durum wheat strengthens the possibility of developing these BCAs as commercial products.

## 4. Materials and Methods

### 4.1. Salicylic and Jasmonic Acid in Wheat Spikes Inoculated with B. Velezensis RC 218 and F. Graminearum under Greenhouse Conditions

#### 4.1.1. Greenhouse Conditions and Treatments Application

Five wheat seedlings (variety BioINTA 1005-susceptible to *F. graminearum*) in a 24-cm-diameter pot (containing soil, sand and river peat mixed in equal parts) were grown under greenhouse conditions for 12 weeks prior to inoculation. A bioformulate of *B. velezensis* RC 218 (described in Section 4.2.2) and *F. graminearum* RC 276 [16] were used to carry out the experiments. The treatments applied at anthesis stage were: 1) *F. graminearum* alone (5 mL); 2) the bioformulated suspension and *F. graminearum* (5 mL + 5 mL); 3) the bioformulated suspension (5 mL); and 4) sterile distilled water and Tween 80 (0.05% *v v*^−1^) (5 mL). Inoculated spikes were maintained at 90% humidity with automatic foggers for three days to ensure pathogen colonization. In order to evaluate JA and SA profile production under the different treatments, spike sampling was performed at 0, 6, 12, 24, 48 and 72 h post inoculation. Each sample consisted of three spikes of each treatment at each sampling period. Treatments details are shown in Table 2 and were done in triplicate for two years.

#### 4.1.2. Phytohormone Extraction and Quantification by High Performance Liquid Chromatography-Tandem Mass Spectrometry

In each sampling period, sampled spikes were immediately frozen in liquid nitrogen, milled in a mortar and stored at −80 °C until phytohormone extraction was done. Milled spike tissue (200 mg) was homogenized in 5 mL bi-distilled water and supplemented with 20 µL of a mixture of internal standards containing 50 ng [2H4]-SA and 50 ng [2H6]-JA. Centrifugation was performed at 5000 rpm for 15 min, the pellet was discarded, the pH of the supernatant was adjusted to 2.8 with acetic acid, and the supernatant was partitioned twice against an equal volume of diethyl ether [38]. The aqueous phase was discarded, and the organic fraction was evaporated. For the quantification of JA and SA, the samples were resuspended in 100 μL of acetonitrile/water (20:80, *v*/*v*) containing 0.3 mmol/L NH_4_HCOO (adjusted to pH 3.5 with formic acid). Afterwards, they were sonicated for 5 min and centrifuged at 16,000× *g* for 5 min. For analysis, the supernatant was 10-fold diluted in acetonitrile/water (20:80, *v*/*v*) containing 0.3 mmol/L NH_4_HCOO (adjusted to pH 3.5 with formic acid). Reversed phase separation of constituents was achieved by Ultra Performance Liquid Chromatography using an ACQUITY UPLC^®^ system (Waters Corp., Milford, MA, USA) equipped with an ACQUITY UPLC^®^ High Strength Silica T3 column (100 mm × 1 mm, 1.8 µm; Waters Corp., Milford, MA, USA). Aliquots of 10 µL were injected in a partial loop with needle overfill mode. Elution was adapted to Balcke et al. [39]. Solvent A and B were water and acetonitrile/water (90:10, *v*/*v*), respectively, both containing 0.3 mmol/L NH_4_HCOO (adjusted to pH 3.5 with formic acid). The flow rate was 0.16 mL/min and the separation temperature was constantly at 40 °C. Elution was performed isocratically for 0.5 min at 10% solution B, followed by a linear increase to 40% solution B in 1.5 min, this condition was held for 2 min, followed by a linear increase to 95% solution B in 1 min, this condition was held for 2.5 min. The column was re-equilibrated for start conditions in 3 min.

Nanoelectrospray (nanoESI) analysis was achieved using a chip ion source (TriVersa Nanomate^®^; Advion BioSciences, Ithaca, NY, USA). For stable nanoESI, 70 µL min^−1^ of 2-propanol/acetonitrile/water (70:20:10, *v*/*v*/*v*) containing 0.3 mmol/L NH_4_HCOO (adjusted to pH 3.5 with formic acid) delivered by a Pharmacia 2248 HPLC pump (GE Healthcare, Munich, Germany) were added just after the column via a mixing tee valve. By using another post column splitter, 502 nL min^−1^ of the eluent were directed to the nanoESI chip with 5 µm internal diameter nozzles. The ionization voltage was set to −1.7 kV. Phytohormones were ionized in a negative mode and determined in a scheduled multiple reaction monitoring mode with an AB Sciex 4000 QTRAP^®^ tandem mass spectrometer (AB Sciex, Framingham, MA, USA). Mass transitions were as follows: 215/59 (declustering potential (DP) −35 V, entrance potential (EP) −8.5 V, collision energy (CE) −24 V) for D6-JA, 209/59 (DP −30 V, EP −4.5 V, CE −24 V) for JA, 141/97 (DP −25 V, EP −6 V, CE −22 V) for D4-SA and 137/93 (DP −25 V, EP −6 V, CE −20 V) for SA. The mass analyzers were adjusted to a resolution of 0.7 amu full width at half-height. The ion source temperature was 40 °C, and the curtain gas was set at 10 (given in arbitrary units) [40].

### 4.2. Evaluation of Bacillus Velezensis RC 218 and Streptomyces Albidoflavus RC 87B as Biocontrol Agents against F. Graminearum in Durum Wheat under Field Conditions

#### 4.2.1. Field Trial on Durum Wheat

The field trial was carried out in Necochea, Buenos Aires province, Argentina, during the 2016/17 harvest season. The durum wheat (*Triticum turgidum L.* var *durum*) cultivar ACA 1901F (susceptible to *F. graminearum*) was sown at the end of July. Biocontrol assays took place from wheat flowering stage (end of October 2016) until crop harvest (mid-January 2017). A complete randomized design was used with three replicates (plots) per treatment. Each experimental plot consisted of three rows (2 m/row, 0.2 m between rows; 250 head per plot).

#### 4.2.2. Culture Conditions of BCAs and Pathogen Production

The *Bacillus velezensis* RC 218 and *S. albidoflavus* RC 87B used in this study were originally isolated from wheat anthers as potential biocontrol agents against *F. graminearum* in Argentina [16,20,26]. These strains are maintained in the culture collection Department of Microbiology and Immunology at the Universidad Nacional de Río Cuarto; Río Cuarto, Córdoba, Argentina). Biomass of *B. velezensis* RC 218 and *S. albidoflavus* RC 87B was produced in liquid basic medium (sucrose 10 g/L, yeast extract 5 g/L) as was described by Costa et al. [41], incubated for 48 h at 28 °C in a rotatory shaker (150 rpm). For *B. velezensis* RC 218 biomass production, the liquid media was modified with NaCl (water activity of 0.97) in order to obtain a physiological improvement of the strain by the intracellular accumulation of betaine [33]. After biomass production, cells were centrifuged at 10,000 rpm for 10 min, washed with sterile distilled water, centrifuged again and, finally, resuspended in sterile distilled water with the addition of a protectant. Finally, the *B. velezensis* RC 218 bioformulate was freeze-dried. *Streptomyces albidoflavus* RC 87B biomass was centrifuged as above and used as fresh cells. The viability of the biocontrol agents was evaluated by plate counting on agarized basic medium and adjusted to 1 × 10^6^ colony-forming units per mL (cfu mL^−1^). *Fusarium graminearum* strains S-5 and S-17 were used as an inoculum mixture to inoculate the durum wheat at the anthesis stage. The toxigenic profile of the strains was determined in previous studies [42]. *Fusarium graminearum* conidia were produced in Mung bean broth [43] and after seven days of incubation at 25 °C and 200 rpm on a rotatory shaker, cultures were centrifuged (7000 rpm; 5 min), resuspended in sterile distilled water plus Tween 80 (0.05%) and filtered through sterile gauze to obtain the macroconidia suspension. Macroconidia counting was performed in a haemocytometer chamber and the concentration was adjusted to 5 × 10^5^ conidia mL^−1^ (1:1 mixture S-5 and S-17 strains).

#### 4.2.3. Biocontrol Agents and Pathogen Applications

The application of the BCAs and the *F. graminearum* mixture inoculum was done at the anthesis stage period, when 50% of the heads in the plots were at the flowering stage (Feekes stage 10.5.2–10.5.3) [44]. Treatments are shown in Table 3. Before treatment applications, wheat heads were always misted with water for 2 min in order to increase its humidity. Either *B. velezensis* RC 218 or *S. albidoflavus* RC 87B were applied before *F. graminearum* inoculum mixture. Treatments were applied by using a commercial CO_2_ sprayer (30 psi) consisting of three linear sprinklers (Teejet twinjet TJ-60-6502) at a rate of 15 mL per linear meter. Positive control plots were only inoculated with the *F. graminearum* strain mixture and negative control plots were inoculated with sterile distilled water plus Tween 80 (0.05%). All treatments were done in triplicate. Humidity in the field was achieved using water sprinklers, and were turned on for 30 min every 2 h from 8 a.m. to 6 p.m. during the three days after inoculations.

#### 4.2.4. Fusarium Head Blight Incidence and Severity, DON Content, *F. graminearum* DNA Levels by qPCR and *Fusarium*-Damaged Kernels on Harvested Grains

FHB disease incidence and severity were evaluated 21 days after inoculations. FHB incidence was determined by counting symptomatic spikes and divided by the total spikes of the plot (treatment replicate); disease severity was evaluated by observing symptomatic spikelets (decoloured, browny) and visually compared with a 0–100% scale [45].

At the harvest time, wheat heads were collected to determine the DON concentration in the grains. Toxin extraction was done by mixing 15 g of milled grains and acetonitrile:water (84:16, 100 mL), shaken for 30 min, filtered by Whatman N°1 and 5 mL of the filtrated was passed through a clean-up column (Mycosep 225, Romer, Sandy Drive, DE, USA). Then, 2 mL of the filtrate was evaporated to dryness (N_2_, 50 °C). DON concentration was determined by liquid chromatography as previously described [15]. Briefly, the HPLC system consisted of a Hewlett Packard model 1100 pump (Palo Alto, CA, USA) connected to a Hewlett Packard 1100 Series variable wavelength detector and a data module Hewlett Packard Kayak XA (HP ChemStation Rev. A.06.01, Palo Alto, CA, USA). Chromatographic separations were performed on a Luna™ C18 reversed-phase column (100 × 4.6 mm, 5 µm particle size, Phenomenex, Torrance, CA, USA) connected to a guard column SecurityGuard™ (Phenomenex, Torrance, CA, USA) (4 × 3.0 mm) filled with the same phase. The mobile phase consisted of methanol/water (12:88, *v*/*v*), at a flow rate of 1.5 mL min^−1^. The detector was set at 220 nm with an attenuation of 0.01 Absorbance Units Full Scale (AUFS). The injection volume was 50 µL and the retention time of DON was 800 s. Quantification was relative to external standards of 1 to 4 µg mL^−1^ in methanol/water (5:95). The quantification limit was 0.5 µg g^−1^.

Grains were pulverized in a mill with a 1 mm^2^ mesh (Cyclotech, Foss Tecator, West Sussex, UK) and total DNA (10 mg) was extracted by using the DNeasy 96 plant kit (Qiagen, Hilden, Germany) according to the manufacturer’s instructions. TaqMan qPCR quantifications were done on an ABI Prism 7500 Sequence Detection System (Applied Biosystems, Lincoln Centre Drive Foster City, CA, USA). For *F. graminearum* DNA quantification, separate TaqMan reactions were performed in 25 µL, using 12.5 µL of TaqMan 2X universal PCR master mix (Applied Biosystems) and 100 nM of FAM-labeled probe and internal control probe and 400 nM of forward and reverse primer for *F. graminearum* target as well as an internal positive control were performed. Primers and probes information were described in Palazzini et al. (2013) [20]. qPCR reactions were performed on 2 µL of DNA preparations from grain samples and pure pathogen DNA. Thermal cycling conditions consisted of a single cycle of 10 min at 95 °C, followed by 40 cycles of 95 °C for 15 s and 60 °C for 1 min. A standard curve was generated by using 10-fold serial dilutions of pure *F. graminearum* DNA in the range of 0.1 pg µL^−1^ to 1 × 10^4^ pg µL^−1^ and pathogen quantifications were done by quintuplicate. During samples analyses, samples of the pathogen DNA were run in parallel using a 10-fold serial dilutions ranging 0.1 pg µL^−1^ to 1 × 10^4^ pg µL^−1^ as reference. From the obtained Ct-values of qPCR for the pathogen DNA dilution series and for DNA extracts from wheat grain samples, the concentrations of *F. graminearum* DNA in the samples were calculated, expressed as pg of DNA of fungal pathogen per mg of grain.

*Fusarium*-damaged kernels (FDK) is another common parameter used as an indicator of disease severity. A total of 300 grains per treatment were analyzed and FDK was defined as the percentage of scabby grains (shriveled, pink-to-white grains, discolored) present in the sample.

#### 4.2.5. Statistical Analyses of Phytohormone Values and FHB Parameters Evaluated

Phytohormone’s data were subjected to one way analysis of variance (ANOVA) and means were separated by Tukey’s method (*p* ≤ 0.05). FHB disease severity were subjected to ANOVA on ranks and means were separated by Dunn’s method (*p* ≤ 0.001). Deoxynivalenol accumulation data were subjected to a one way ANOVA and means were separated by Duncan’s method (*p* ≤ 0.05). DNA quantification and FDK data were subjected to a one way ANOVA and means were separated by the Fisher and Holm–Sidak methods, respectively (*p* ≤ 0.05). All statistical analyses were performed using SigmaStat for Windows version 3.5 (SPSS Inc., Chicago, IL, USA, 2007).

## Figures and Tables

**Table 1:** *Fusarium graminearum* DNA content, *Fusarium*-damaged kernels and deoxynivalenol accumulation on harvested grains.

Treatments	Fusarium Head Blight Parameters		
	*F. graminearum* DNA (pg DNA mg grain^−1^) *	*Fusarium*-Damaged Kernels	Deoxynivalenol (µg g^−1^)
*F. graminearum* control	60.7 ± 24.7a	10.88 ± 2.2a	4.43 ± 1.7a
*B. velezensis* RC 218 + *F. graminearum*	23.8 ± 11.1b	6 ± 0.58b	2.2 ± 0.5b
*S. albidoflavus* RC 87B + *F. graminearum*	37.1 ± 22.7ab	7 ± 2.36b	2.18 ± 0.16b
*B. velezensis* RC 218	2.6 ± 1.04b	5.22 ± 2.5b	Nd
*S. albidoflavus* RC 87B	7.6 ± 0.4b	3.44 ± 0.38b	Nd
Water Control	10.1 ± 0.03b	5.5 ± 3.47b	0.3 ± 0.13bc

* *F. graminearum* DNA means were separated using Fisher’s method (*p* ≤ 0.05); *Fusarium* Damaged Kernels means were separated by the Holm–Sidak method (*p* ≤ 0.05) and deoxynivalenol means were separated using Duncan’s method (*p* ≤ 0.05). On each column, values with different letters indicate significant differences. Nd: Not detected, detection limit 0.5 µg g^−1^.

**Table 2:** Treatments evaluated in the phytohormones’ greenhouse experiment.

Treatments	Inoculum Concentration
*F. graminearum* control	1 × 10^5^ conidia mL^−1^
*B. velezensis* RC 218 + *F. graminearum*	1 × 10^6^ cfu mL^−1^ + 1 × 10^5^ conidia mL^−1^
*B. velezensis* RC 218 control	1 × 10^6^ cfu mL^−1^
Water Control	-*

* Water control consisted of plots inoculated with sterile distilled water plus Tween 80 (0.05%).

**Table 3:** Treatments evaluated in the durum wheat field experiment.

Treatments	Inoculum Concentration
*F. graminearum* control	1 × 10^5^ conidia mL^−1^
*B. velezensis* RC 218 + *F. graminearum*	1 × 10^6^ cfu mL^−1^ + 1 × 10^5^ conidia mL^−1^
*S. albidoflavus* RC 87B + *F. graminearum*	1 × 10^6^ cfu mL^−1^ + 1 × 10^5^ conidia mL^−1^
*B. velezensis* RC 218 control	1 × 10^6^ cfu mL^−1^
*S. albidoflavus* RC 87B control	1 × 10^6^ cfu mL^−1^
Water Control	-*

* Water control consisted of plots inoculated with sterile distilled water plus Tween 80 (0.05%).
